# Circulating osteoprotegerin levels and cardiovascular outcomes in patients with pre-dialysis chronic kidney disease: results from the KNOW-CKD study

**DOI:** 10.1038/s41598-024-54335-y

**Published:** 2024-02-19

**Authors:** Sang Heon Suh, Tae Ryom Oh, Hong Sang Choi, Chang Seong Kim, Eun Hui Bae, Seong Kwon Ma, Kook-Hwan Oh, Kyu-Beck Lee, Jong Cheol Jeong, Ji Yong Jung, Soo Wan Kim, The Korean Cohort Study for Outcomes in Patients With Chronic Kidney Disease (KNOW-CKD) Investigators

**Affiliations:** 1 Department of Internal Medicine, Chonnam National University Medical School and Chonnam National University Hospital, 42 Jebongro, Gwangju, 61469 Korea; 2 Department of Internal Medicine, Seoul National University Hospital, Seoul, Korea; 3 Division of Nephrology, Department of Internal Medicine, Kangbuk Samsung Hospital, Sungkyunkwan University School of Medicine, Seoul, South Korea; 4 Division of Nephrology, Department of Internal Medicine, Seoul National University Bundang Hospital, Seongnam, Republic of Korea; 5 Division of Nephrology, Department of Internal Medicine, Gachon University Gil Medical Center, Incheon, Republic of Korea

**Keywords:** Biomarker, Chronic kidney disease, Major adverse cardiac event, Osteoprotegerin, Kidney diseases, Cardiovascular biology

## Abstract

While the relationship between circulating osteoprotegerin (OPG) and cardiovascular events is well-established in the general population, its association with cardiovascular risks in chronic kidney disease (CKD) patients remains less robust. This study hypothesized that elevated circulating OPG levels might be associated with an increased risk of major adverse cardiac events (MACE) in CKD patients, a total of 2,109 patients with CKD stages 1 through pre-dialysis 5 from the KNOW-CKD cohort were categorized into quartiles based on serum OPG levels. The primary outcome of the study was 3-point MACE, defined as a composite of nonfatal myocardial infarction, nonfatal stroke, or cardiac death. The median follow-up duration was 7.9 years. The cumulative incidence of 3-point MACE significantly varied across serum OPG levels in Kaplan–Meier curve analysis (*P* < 0.001, log-rank test), with the highest incidence observed in the 4th quartile. Cox regression analysis indicated that, relative to the 1st quartile, the risk of 3-point MACE was significantly higher in the 3rd (adjusted hazard ratio 2.901, 95% confidence interval 1.009 to 8.341) and the 4th quartiles (adjusted hazard ratio 4.347, 95% confidence interval 1.410 to 13.395). In conclusion, elevated circulating OPG levels are associated with adverse cardiovascular outcomes in pre-dialysis CKD patients.

## Introduction

Cardiovascular disease (CVD) remains a primary cause of mortality among patients with chronic kidney disease (CKD)^1,2^. Beyond the notable prevalence of coronary artery disease^3,4^, heart failure, stemming from either systolic or diastolic dysfunction, further exacerbates the adverse cardiovascular outcomes in this population^5,6^. Hence, the early stratification of CKD patients at heightened risk for cardiovascular events (CVEs) is crucial. Several biomarkers have been explored for their potential to predict forthcoming CVEs in the CKD cohort^7–9^; however, none has unequivocally outperformed the rest.

Osteoprotegerin (OPG), a secreted glycoprotein, is a member of the tumor necrosis factor receptor (TNF) superfamily^10,11^. While primarily known for its role in bone metabolism—it mediates osteoclastogenesis inhibition by binding to the receptor activator of nuclear factor-κB ligand (RANKL) and preventing the RANKL-RANK interaction^10–12^—circulating OPG’s clinical significance extends beyond bone health. Accumulating evidence underscores a robust correlation between elevated circulating OPG levels and heightened CVE risk^13^. Multiple studies have consistently linked increased circulating OPG concentrations with adverse CVE outcomes in patients diagnosed with CAD^14–16^. The predictive capacity of OPG in determining coronary artery calcification risk is further corroborated by meta-analyses^17,18^. Moreover, the CORONA trial’s post-hoc analysis affirmed the relationship between serum OPG concentrations and the exacerbation of heart failure^19^. Therefore, the prognostic utility of circulating OPG concerning CVEs in the broader population is well-established.

Despite the observed elevation in serum OPG levels among patients with CKD^20^, the evidence substantiating the association between circulating OPG and CVEs remains less robust than in the general population. Previous studies have proposed an association between circulating OPG levels and vascular calcification^21^, myocardial dysfunction^22^, cardiovascular mortality^23,24^, and all-cause mortality^25^. However, these studies are limited by their small sample sizes and relatively short follow-up durations. Furthermore, the evidence connecting serum OPG levels to overarching cardiovascular outcomes, particularly the risk of major adverse cardiac events (MACE), remains scant in the CKD patient cohort.

In the present study, utilizing a long-term prospective cohort of patients with CKD ranging from stage 1 to pre-dialysis stage 5, we sought to elucidate the association between circulating OPG levels and the risk of CVEs. Our hypothesis posits that elevated circulating OPG levels might correlate with a heightened risk of adverse CVEs in CKD patients.

## Results

### Baseline characteristics

Significant differences in serum OPG levels among participants were observed (Table 1). The shortest follow-up duration was noted in Q4. Additionally, the mean age was highest in Q4. There was a higher proportion of subjects with a Charlson comorbidity index ≥ 4 and those with DM in Q4. The frequency of diuretics, statins, and antiplatelet and/or anticoagulant usage was also higher in Q4. Waist-to-hip ratio (WHR) and blood pressure levels were notably elevated in Q4, whereas levels of hemoglobin, albumin, total cholesterol, high-density lipoprotein cholesterol (HDL-C), and estimated glomerular filtration rate (eGFR) were reduced. Conversely, high-sensitivity C-reactive protein (hs-CRP) and spot urine albumin-to-creatinine ratio (ACR) levels were increased in Q4. Echocardiographic data revealed an association between increased serum OPG levels and elevations in left ventricular mass index, E/e′ ratio, left atrium diameter, interventricular wall thickness, and posterior wall thickness (Supplementary Table S1). Moreover, the prevalence of regional wall motion abnormality and valvular calcification was higher in Q4. Conversely, left ventricular ejection fraction (LVEF) was diminished in Q4. In essence, adverse medical conditions frequently correlated with elevated serum OPG levels.

**Table 1:** Baseline characteristics of study participants by serum OPG levels.

	Serum OPG levels				*P* value
	Q1	Q2	Q3	Q4	
Follow-up duration (years)	7.705 ± 2.587	7.426 ± 2.668	6.983 ± 2.997	6.444 ± 3.094	< 0.001
Age (years)	43.859 ± 10.898	51.338 ± 10.734	56.309 ± 10.463	62.672 ± 8.186	< 0.001
Male	185 (35.2)	223 (42.2)	216 (40.9)	199 (37.8)	0.090
Charlson comorbidity index					< 0.001
0–3	496 (94.5)	436 (82.4)	357 (67.6)	208 (39.5)	
4–5	29 (5.5)	90 (17.0)	156 (29.5)	301 (57.1)	
6–7	0 (0.0)	3 (0.6)	15 (2.8)	17 (3.2)	
≥ 8	0 (0.0)	0 (0.0)	0 (0.0)	1 (0.2)	
Primary renal disease					< 0.001
DM	25 (4.8)	87 (16.4)	143 (27.2)	277 (52.6)	
HTN	91 (17.3)	104 (19.7)	129 (24.5)	94 (17.8)	
GN	247 (47.0)	197 (37.2)	149 (28.3)	72 (13.7)	
TID	4 (0.8)	2 (0.4)	3 (0.6)	5 (0.9)	
PKD	124 (23.6)	105 (19.8)	75 (14.3)	36 (6.8)	
Others	34 (6.5)	34 (6.4)	27 (5.1)	43 (8.2)	
Smoking status					0.056
Non-smoker	285 (54.3)	284 (53.8)	284 (54.0)	269 (51.0)	
Ex-smoker	98 (18.7)	92 (17.4)	69 (13.1)	79 (15.0)	
Current smoker	142 (27.0)	152 (28.8)	173 (32.9)	179 (34.0)	
Medication					
ACEIs/ARBs	455 (86.7)	455 (86.2)	456 (86.5)	436 (82.7)	0.214
Diuretics	103 (19.6)	144 (27.3)	180 (34.2)	246 (46.7)	< 0.001
Statins	221 (42.1)	265 (50.2)	303 (57.5)	308 (58.4)	< 0.001
Antiplatelets/anticoagulants	90 (17.1)	141 (26.7)	164 (31.1)	202 (38.3)	< 0.001
BMI (kg/m^2^)	24.618 ± 3.600	24.604 ± 3.473	24.659 ± 3.417	24.414 ± 3.085	0.607
WHR	0.883 ± 0.067	0.893 ± 0.063	0.904 ± 0.065	0.914 ± 0.063	< 0.001
SBP (mmHg)	124.368 ± 14.616	126.602 ± 14.324	127.472 ± 15.673	132.608 ± 18.619	< 0.001
DBP (mmHg)	77.663 ± 10.787	78.146 ± 10.454	76.442 ± 10.829	75.759 ± 12.247	0.002
Laboratory findings					
Hemoglobin (g/dL)	13.797 ± 1.848	13.174 ± 1.927	12.713 ± 1.907	11.654 ± 1.776	< 0.001
Albumin (g/dL)	4.303 ± 0.347	4.227 ± 0.385	4.179 ± 0.403	3.987 ± 0.496	< 0.001
Total cholesterol (mg/dL)	175.326 ± 33.617	176.053 ± 39.909	174.918 ± 42.788	170.998 ± 40.228	0.164
HDL-C (mg/dL)	50.442 ± 15.036	51.409 ± 15.771	48.723 ± 15.515	46.864 ± 15.464	< 0.001
LDL-C (mg/dL)	98.451 ± 28.526	98.528 ± 33.035	96.324 ± 34.004	94.441 ± 31.521	0.109
TG (mg/dL)	155.374 ± 97.262	147.515 ± 83.379	166.666 ± 113.581	161.097 ± 100.508	0.011
Fasting glucose (mg/dL)	101.237 ± 22.014	105.522 ± 30.181	113.065 ± 40.451	123.380 ± 54.767	< 0.001
25(OH)D	18.479 ± 7.324	18.154 ± 7.156	17.813 ± 7.318	17.115 ± 9.582	0.064
hs-CRP (mg/dL)	0.500 [0.200, 1.300]	0.680 [0.300, 1.550]	0.695 [0.300, 1.800]	0.700 [0.230, 2.100]	0.034
Spot urine ACR (mg/g)	197.628 [32.628, 599.759]	293.761 [52.457, 761.677]	391.228 [92.824, 1106.630]	716.078 [198.801, 1926.921]	< 0.001
Creatinine (mg/dL)	1.378 ± 0.966	1.567 ± 0.920	1.856 ± 1.052	2.481 ± 1.321	< 0.001
eGFR (mL/min./1.73 m^2^)	67.953 ± 32.409	56.806 ± 30.766	45.015 ± 24.112	31.637 ± 18.399	< 0.001
CKD stages					< 0.001
Stage 1	175 (33.3)	108 (20.4)	45 (8.5)	12 (2.3)	
Stage 2	146 (27.8)	130 (24.6)	92 (17.4)	30 (5.7)	
Stage 3a	88 (16.8)	91 (17.2)	102 (19.3)	65 (12.3)	
Stage 3b	68 (13.0)	107 (20.2)	142 (26.9)	126 (23.9)	
Stage 4	41 (7.8)	77 (14.6)	120 (22.7)	214 (40.6)	
Stage 5	7 (1.3)	16 (3.0)	27 (5.1)	80 (15.2)	

Values for categorical variables are given as number (percentage); values for continuous variables, as mean ± standard deviation or median [interquartile range]. *25(OH)D* 25-hydroxyvitamin D, *ACEIs* angiotensin converting enzyme inhibitors, *ACR* albumin-to-creatinine ratio, *ARBs* angiotensin receptor blockers, *BMI* body mass index, *CKD* chronic kidney disease, *DBP* diastolic blood pressure, *DM* diabetes mellitus, *eGFR* estimated glomerular filtration rate, *GN* glomerulonephritis, *HDL-C* high-density lipoprotein cholesterol, *hs-CRP* high-sensitivity C-reactive protein, *HTN* hypertension, *LDL-C* low-density lipoprotein cholesterol, *OPG* osteoprotegerin, *PKD* polycystic kidney disease, *Q1* 1st quartile, *Q2* 2nd quartile, *Q3* 3rd quartile, *Q4* 4th quartile, *SBP* systolic blood pressure, *TG* triglyceride, *TID* tubulointerstitial disease, *WHR* waist-to-hip ratio.

### Association between serum OPG levels and the risk of MACEs in patients with CKD

**Figure 1:**
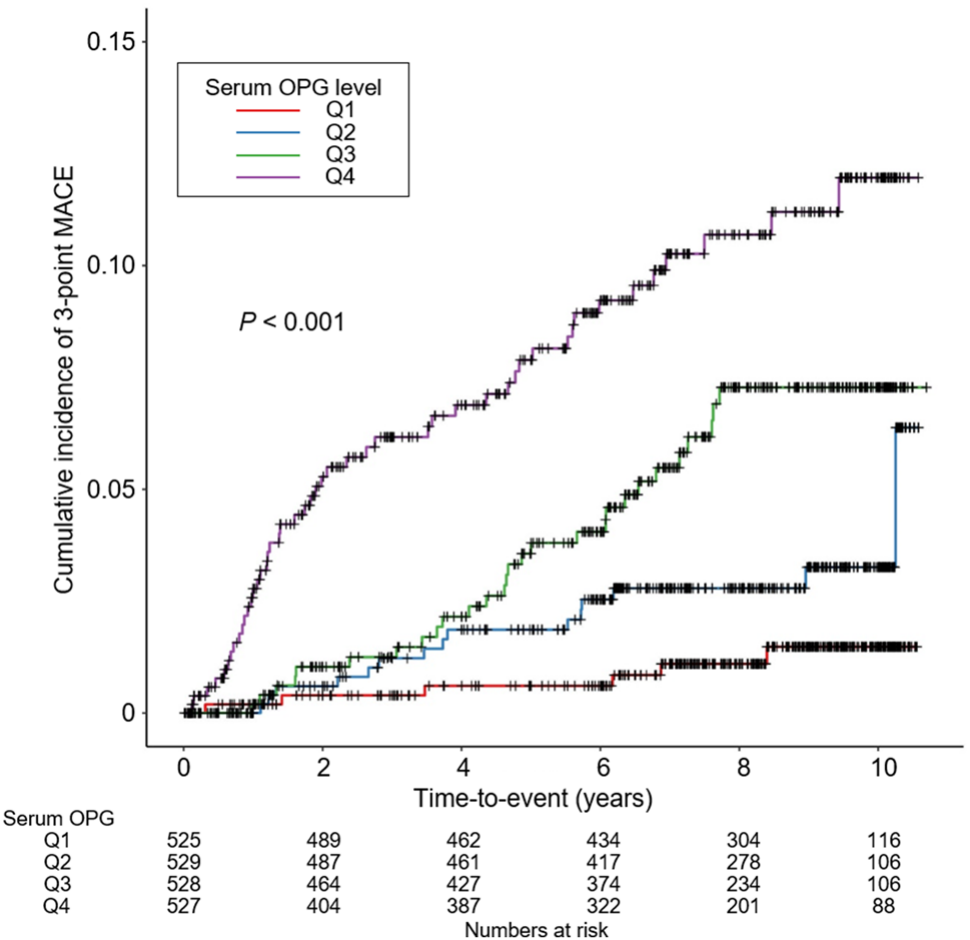
Kaplan–Meier survival curve for cumulative incidence of 3-point MACE by serum OPG levels. *P* value determined by log-rank test. *MACE* major adverse cardiac event, *OPG* osteoprotegerin, *Q1* 1st quartile, *Q2* 2nd quartile, *Q3* 3rd quartile, *Q4* 4th quartile.

**Figure 2:**
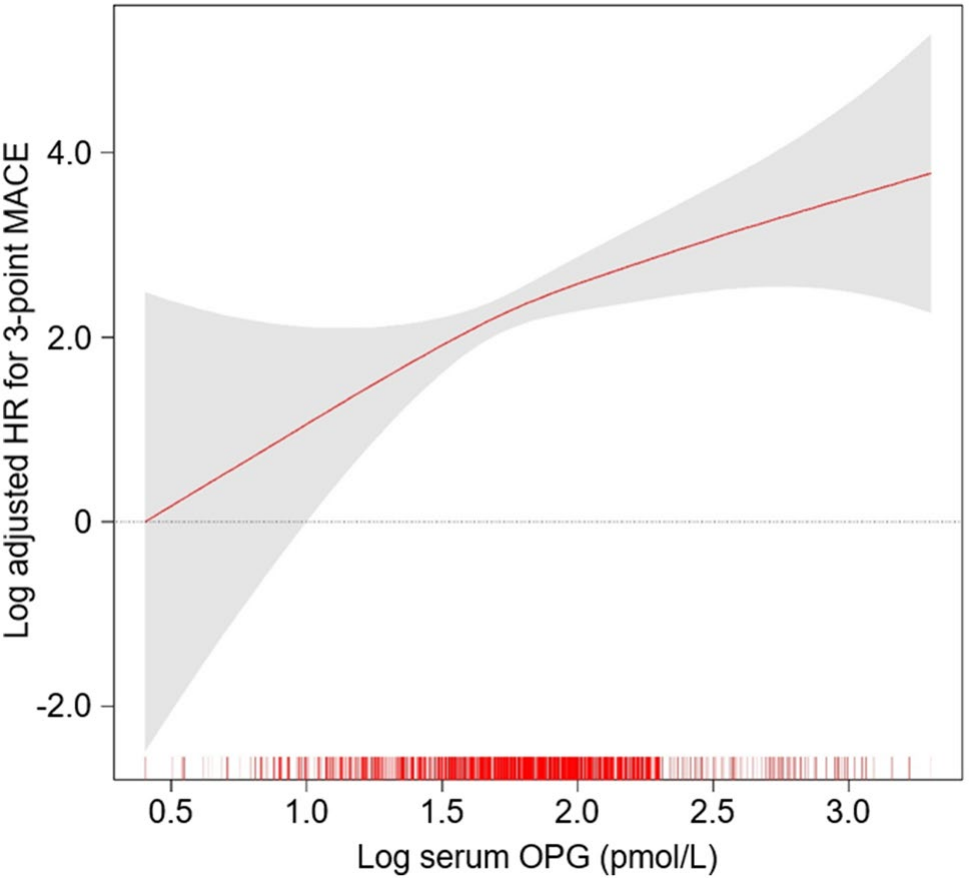
Panelized spline curve of serum OPG level’s effect on 3-point MACE. The adjusted HR for serum OPG level, considered as a continuous variable for 3-point MACE, is depicted. This model was adjusted for factors including age, sex, Charlson comorbidity index, primary causes of CKD, smoking status, medications (ACEIs/ARBs, diuretics, statins, antiplatelets/anticoagulants), WHR, SBP, hemoglobin, albumin, total cholesterol, HDL-C, fasting glucose, 25(OH)D, hs-CRP, eGFR, spot urine ACR, and LVEF at baseline. *HR* hazard ratio, *MACE* major adverse cardiac event, *OPG* osteoprotegerin.

The study outcomes’ cumulative incidence was evaluated using the Kaplan–Meier curve. The risks associated with incident 3-point (Fig. 1), 4-point (Supplementary Fig. S1), and 6-point (Supplementary Fig. S2) MACEs varied significantly based on serum OPG levels, with the highest incidence observed in Q4 (all *P* < 0.001). Cox proportional hazard models were utilized to determine the independent association between serum OPG levels and MACE risks. Relative to Q1, there was a significant elevation in the risk of 3-point MACE in Q3 (adjusted HR 2.901, 95% CI 1.009–8.341) and Q4 (adjusted HR 4.347, 95% CI 1.410–13.395) as presented in Table 2. Additionally, the risks for 4-point (adjusted HR 2.571, 95% CI 1.006–6.570) and 6-point (adjusted HR 2.686, 95% CI 1.179–6.116) MACEs were notably higher in Q4 compared to Q1 (Supplementary Table S2). Spline curve analyses further illustrated a nearly linear, positive correlation between serum OPG levels and MACE risk (Fig. 2 and Supplementary Figs. S3 and S4).

**Table 2:** HRs for 3-point MACE by serum OPG levels.

Serum OPG levels (pmol/L)		Events, n (%)	Model 1		Model 2		Model 3		Model 4	
			HR (95% CI)	*P* value	HR (95% CI)	*P* value	HR (95% CI)	*P* value	HR (95% CI)	*P* value
3-point MACE										
Q1	1.5–4.5	6 (1.1)	Reference		Reference		Reference		Reference	
Q2	4.5–6.0	15 (2.8)	2.889 (1.041, 8.022)	0.042	1.978 (0.755, 5.18)	0.165	1.670 (0.626, 4.457)	0.306	2.097 (0.732, 6.006)	0.168
Q3	6.0–8.2	28 (5.3)	5.068 (1.926, 13.333)	0.001	3.325 (1.321, 8.372)	0.011	2.402 (0.92, 6.27)	0.073	2.901 (1.009, 8.341)	0.048
Q4	8.2–44.2	48 (9.1)	10.064 (3.974, 25.486)	< 0.001	4.992 (1.967, 12.669)	< 0.001	3.572 (1.326, 9.623)	0.012	4.347 (1.410, 13.395)	0.011

Model 1, unadjusted model. Model 2, model 1 + adjusted for age and sex. Model 3, model 2 + Charlson comorbidity index, primary cause of CKD, smoking status, medication (ACEIs/ARBs, diuretics, statins and antiplatelets/anticoagulants), WHR, and SBP. Model 4, model 3 + adjusted for hemoglobin, albumin, total cholesterol, HDL-C, fasting glucose, 25(OH)D, hs-CRP, eGFR, spot urine ACR, and LVEF at the baseline. *CI* confidence interval, *HR* hazard ratio, *MACE* major adverse cardiac event, *OPG* osteoprotegerin, *Q1* 1st quartile, *Q2* 2nd quartile, *Q3* 3rd quartile, *Q4* 4th quartile.

### Sensitivity and subgroup analyses

Upon re-categorizing participants based on tertile and quintile divisions of serum OPG levels, as opposed to quartile divisions, a significant association between elevated serum OPG levels and an increased risk of 3-point MACE was observed. Specifically, the 3rd tertile versus the 1st tertile yielded an adjusted HR of 3.228 (95% CI 1.228–8.484), and the 5th quintile versus the 1st quintile resulted in an adjusted HR of 4.079 (95% CI 1.177–14.134) (Supplementary Table S3). When non-cardiac death events were censored prior to achieving the study outcome, the increased risk associated with 3-point MACE remained significant in the 4th quartile with an adjusted HR of 4.347 (95% CI 1.212–15.592) (Table 3). Further, after imputing missing values through multiple imputation techniques, the correlation between serum OPG levels and the 3-point MACE risk persisted (4th quartile vs. 1st quartile, adjusted HR 3.239, 95% CI 1.150–9.124) (Table 4). The subsequent subgroup analyses indicated that this association was not significantly influenced by variables such as age, sex, presence or absence of DM, BMI, eGFR, or albuminuria (Table 5).

**Table 3:** Cause-specific HRs for 3-point MACE by serum OPG levels.

Serum OPG levels (pmol/L)	Model 1		Model 2		Model 3		Model 4	
	HR (95% CI)	*P* value	HR (95% CI)	*P* value	HR (95% CI)	*P* value	HR (95% CI)	*P* value
3-point MACE								
Q1	Reference		Reference		Reference		Reference	
Q2	2.580 (1.004, 6.629)	0.049	1.978 (0.736, 5.313)	0.176	1.670 (0.590, 4.710)	0.333	2.096 (0.695, 6.321)	0.189
Q3	5.187 (2.150, 12.514)	< 0.001	3.325 (1.256, 8.800)	0.016	2.402 (0.842, 6.851)	0.101	2.900 (0.923, 9.114)	0.068
Q4	9.801 (4.203, 22.855)	< 0.001	4.992 (1.802, 13.825)	0.002	3.572 (1.157, 11.030)	0.027	4.347 (1.212, 15.592)	0.024

Note: Model 1, unadjusted model. Model 2, model 1 + adjusted for age and sex. Model 3, model 2 + Charlson comorbidity index, primary cause of CKD, smoking status, medication (ACEIs/ARBs, diuretics, statins and antiplatelets/anticoagulants), WHR, and SBP. Model 4, model 3 + adjusted for hemoglobin, albumin, total cholesterol, HDL-C, fasting glucose, 25(OH)D, hs-CRP, eGFR, spot urine ACR, and LVEF at the baseline. *CI* confidence interval, *HR* hazard ratio, *MACE* major adverse cardiac event, *OPG* osteoprotegerin, *Q1* 1st quartile, *Q2* 2nd quartile, *Q3* 3rd quartile, *Q4* 4th quartile.

**Table 4:** HRs for 3-point MACE by serum OPG levels following multiple imputation.

Serum OPG levels (pmol/L)	Model 1		Model 2		Model 3		Model 4	
	HR (95% CI)	*P* value	HR (95% CI)	*P* value	HR (95% CI)	*P* value	HR (95% CI)	*P* value
3-point MACE								
Q1	Reference		Reference		Reference		Reference	
Q2	2.580 (1.001, 6.649)	0.053	1.978 (0.755, 5.180)	0.168	1.703 (0.644, 4.503)	0.286	1.710 (0.642, 4.554)	0.287
Q3	5.187 (2.147, 12.530)	< 0.001	3.325 (1.321, 8.372)	0.012	2.513 (0.974, 6.483)	0.061	2.563 (0.975, 6.734)	0.061
Q4	9.801 (4.192, 22.917)	< 0.001	4.992 (1.967, 12.669)	0.001	3.288 (1.234, 8.763)	0.020	3.239 (1.150, 9.124)	0.030

Model 1, unadjusted model. Model 2, model 1 + adjusted for age and sex. Model 3, model 2 + Charlson comorbidity index, primary cause of CKD, smoking status, medication (ACEIs/ARBs, diuretics, statins and antiplatelets/anticoagulants), WHR, and SBP. Model 4, model 3 + adjusted for hemoglobin, albumin, total cholesterol, HDL-C, fasting glucose, 25(OH)D, hs-CRP, eGFR, spot urine ACR, and LVEF at the baseline. *CI* confidence interval, *HR* hazard ratio, *MACE* major adverse cardiac event, *OPG* osteoprotegerin, *Q1* 1st quartile, *Q2* 2nd quartile, *Q3* 3rd quartile, *Q4* 4th quartile.

**Table 5:** HRs for the 3-point MACE by serum OPG level across various subgroups.

Serum OPG level	Events, n (%)	Unadjusted HR (95% CIs)	*P* for interaction	Adjusted HR (95% CIs)	*P* for interaction
Age < 60 years					
Q1	3 (0.6)	Reference	0.065	Reference	0.483
Q2	9 (2.2)	3.144 (1.121, 8.821)		2.081 (0.691, 6.268)	
Q3	12 (3.9)	4.964 (1.788, 13.785)		2.518 (0.781, 8.112)	
Q4	13 (7.9)	13.965 (5.264, 37.046)		3.920 (1.129, 13.613)	
Age ≥ 60 years					
Q1	3 (7.3)	Reference		Reference	
Q2	6 (5.3)	1.018 (0.368, 2.856)		0.452 (0.137, 1.494)	
Q3	16 (7.2)	1.119 (0.431, 2.906)		0.494 (0.162, 1.508)	
Q4	35 (9.7)	2.364 (0.957, 5.837)		0.474 (0.154, 1.457)	
Male					
Q1	4 (1.2)	Reference	0.668	Reference	0.920
Q2	10 (3.3)	2.947 (1.298, 6.692)		0.896 (0.353, 2.272)	
Q3	21 (6.7)	4.252 (1.932, 9.361)		0.867 (0.344, 2.187)	
Q4	36 (11.0)	13.154 (6.357, 27.220)		1.073 (0.409, 2.814)	
Female					
Q1	2 (1.1)	Reference		Reference	
Q2	5 (2.2)	2.546 (0.514, 12.616)		1.847 (0.341, 10.017)	
Q3	7 (3.2)	6.439 (1.463, 28.333)		2.055 (0.386, 10.951)	
Q4	12 (6.0)	12.418 (2.919, 52.824)		1.485 (0.260, 8.501)	
Non-DM					
Q1	4 (0.8)	Reference	0.783	Reference	0.306
Q2	14 (3.2)	2.769 (1.268, 6.046)		1.193 (0.500, 2.848)	
Q3	13 (3.4)	3.824 (1.777, 8.229)		0.912 (0.366, 2.274)	
Q4	17 (6.8)	10.303 (4.990, 21.273)		0.694 (0.264, 1.821)	
DM					
Q1	2 (8.0)	Reference		Reference	
Q2	1 (1.1)	1.370 (0.160, 11.729)		0.359 (0.036, 3.617)	
Q3	15 (10.5)	3.221 (0.429, 24.202)		0.806 (0.094, 6.935)	
Q4	31 (11.2)	6.576 (0.912, 47.418)		1.092 (0.125, 9.541)	
BMI < 23 kg/m^2^					
Q1	1 (0.6)	Reference	0.724	Reference	0.947
Q2	5 (2.9)	3.297 (0.665, 16.342)		0.811 (0.145, 4.528)	
Q3	10 (6.1)	7.857 (1.786, 34.578)		1.275 (0.243, 6.682)	
Q4	17 (10.3)	20.044 (4.793, 83.814)		0.616 (0.104, 3.633)	
BMI ≥ 23 kg/m^2^					
Q1	5 (1.4)	Reference		Reference	
Q2	10 (2.8)	2.487 (1.096, 5.647)		0.945 (0.373, 2.397)	
Q3	18 (5.0)	3.759 (1.708, 8.275)		0.985 (0.390, 2.491)	
Q4	31 (8.6)	10.674 (5.138, 22.176)		1.172 (0.444, 3.090)	
eGFR ≥ 45 mL/min./1.73 m^2^					
Q1	4 (1.0)	Reference	0.465	Reference	0.292
Q2	10 (3.2)	3.497 (1.246, 9.811)		2.289 (0.684, 7.659)	
Q3	14 (6.5)	3.533 (1.184, 10.543)		1.736 (0.467, 6.457)	
Q4	10 (10.1)	12.993 (4.628, 36.481)		2.705 (0.696, 10.508)	
eGFR < 45 mL/min./1.73 m^2^					
Q1	2 (1.5)	Reference		Reference	
Q2	5 (2.3)	1.558 (0.555, 4.371)		0.514 (0.170, 1.552)	
Q3	14 (4.5)	3.039 (1.184, 7.799)		0.666 (0.235, 1.886)	
Q4	38 (8.9)	6.565 (2.666, 16.162)		0.614 (0.212, 1.778)	
Spot urine ACR < 300 mg/g					
Q1	3 (1.0)	Reference	0.591	Reference	0.867
Q2	8 (3.0)	2.735 (1.039, 7.196)		1.046 (0.338, 3.235)	
Q3	15 (6.6)	4.319 (1.714, 10.882)		1.087 (0.350, 3.379)	
Q4	11 (6.7)	8.908 (3.653, 21.720)		0.911 (0.264, 3.148)	
Spot urine ACR ≥ 300 mg/g					
Q1	3 (1.4)	Reference		Reference	
Q2	7 (2.8)	2.699 (0.880, 8.277)		0.889 (0.272, 2.907)	
Q3	12 (4.2)	4.680 (1.612, 13.580)		0.836 (0.251, 2.789)	
Q4	35 (10.0)	14.339 (5.241, 39.228)		1.192 (0.358, 3.968)	

The model was adjusted for age, sex, Charlson comorbidity index, primary causes of CKD, smoking status, medication (ACEIs/ARBs, diuretics, statins, antiplatelets/anticoagulants), WHR, SBP, hemoglobin, albumin, total cholesterol, HDL-C, fasting glucose, 25(OH)D, hs-CRP, eGFR, spot urine ACR, and LVEF at the baseline. *ACR* albumin-to-creatinine ratio, *BMI* body mass index, *CI* confidence interval, *DM* diabetes mellitus, *eGFR* estimated glomerular filtration rate, *HR* hazard ratio, *OPG* osteoprotegerin, *Q1* 1st quartile, *Q2* 2nd quartile, *Q3* 3rd quartile, *Q4* 4th quartile.

## Discussion

In the present study, we determined that elevated circulating levels of OPG correlate with an increased risk of MACE in pre-dialysis CKD patients. The robustness of this finding is underlined by sensitivity analyses, which encompassed both competing risk analysis and multiple imputation, confirming the association between serum OPG levels and MACE risk. Furthermore, subgroup analyses indicated that this correlation remained consistent irrespective of variables such as age, sex, the presence or absence of DM, BMI, eGFR, and albuminuria.

While previous studies have consistently highlighted the predictive capacity of circulating OPG in forecasting CVEs within the general population^14–18^, such a relationship has yet to be affirmed in patients exhibiting renal dysfunction. For instance, Scialla et al*.* reported that OPG may be risk factors for all-cause and cardiovascular mortality in patients initiating dialysis, but not in patients with pre-dialysis CKD, which analyzed 602 incident dialysis patients for median 3.4 years^26^. Marques et al*.* presented a data supporting the association between serum OPG levels and the risk of cardiovascular mortality in patients with CKD stages 3 to 5 (including those undergoing dialysis), while only a limited numbers of the patients were included (n = 145)^24^. Huang et al*.*, in their meta-analysis, demonstrated that elevated OPG level are associated with an increased risk of cardiovascular death in patients with CKD^27^. In the meta-analysis, a total of 2120 patients from 10 studies included, while most of the patients (n = 1723) were receiving dialysis. Therefore, the evidence for the association between serum OPG levels and the risk of MACEs still remains weak especially in patient with pre-dialysis CKD (Supplementary Table S4). This gap in knowledge carries significant implications, especially considering that CVD is a predominant cause of mortality in the CKD population^1,2^. Notably, there remains a lack of specific biomarkers that outperform others in predicting CVD in this specific demographic^7,8^. Furthermore, it should be emphasized that patients with decreased eGFR tend to exhibit elevated serum OPG levels^20^. As such, the predictability of OPG based on findings from the general populace should be approached with caution. Our results, therefore, introduce pivotal evidence concerning the link between serum OPG levels and MACE risk in CKD patients.

Biochemically, OPG operates as a decoy receptor for RANKL and TNF-related apoptosis-inducing ligand (TRAIL)^10–12^. By interacting with RANKL and TRAIL, OPG impedes their subsequent attachment to RANK. It is a recognized phenomenon that the engagement of RANKL with RANK augments the calcification of vascular smooth muscle cells, predominantly through the activation of nuclear factor-κB^28^. Consequently, OPG’s inherent biological role is presumed to act as a defensive mechanism against vascular calcification. Supporting this assertion, one experimental study reported that deleting the gene responsible for encoding murine OPG, *Tnsf11b*, induced spontaneous vascular calcification in mice and accelerated atherosclerosis in *Apoe*-deficient mice^29,30^. This strongly suggests that the prevalent effect of circulating OPG on vascular tissue is fundamentally protective.

On the other hand, OPG treatment in isolated rodent endothelial cells has been shown to lead to a reduction in nitric oxide generation and an increase in reactive oxygen species production^31^. Furthermore, stimulation of these cells with inflammatory cytokines enhances OPG secretion, which subsequently amplifies the surface expression of adhesion molecules, notably ICAM-1 and VCAM-1^32^. These findings suggest a potential role for OPG in early vascular inflammatory processes. Consequently, elevated circulating OPG levels might serve as indicators of vascular inflammation, potentially forecasting future CVEs.

Beyond vascular calcification and atherosclerosis, heart failure stands as a prominent cardiac manifestation correlated with CKD^1,33^. While there exists substantial evidence linking this manifestation to the general population^34–36^, the association between serum OPG levels and the risk of heart failure in CKD patients still requires further validation. In a recent study, a significant relationship was observed between serum OPG levels and risks associated with left ventricular hypertrophy, as well as systolic and diastolic dysfunction, in CKD patients ranging from stage 3 to pre-dialysis 5^22^. However, the limited sample size (n = 101) poses a significant limitation to this study. Our present research does not negate the potential link between serum OPG levels and heart failure risk, especially considering that one of our secondary outcomes, the 6-point MACE, including hospitalization due to heart failure events. Comprehensive studies on a larger scale are imperative to elucidate the relationship between serum OPG levels and heart failure risk.

We acknowledge several limitations in our study. Firstly, given its observational design, this study cannot establish a causal relationship between serum OPG levels and the risk of MACEs. However, it is important to note that OPG serves as a potential biomarker and is not a therapeutic intervention. Conducting randomized controlled trials with serum OPG levels as a primary intervention is not feasible. Nevertheless, post-hoc analyses of clinical trials have shown an association between serum OPG levels and cardiovascular outcomes in the general population. Secondly, all variables, including serum OPG levels, were measured only at baseline. Despite this, many previous studies investigating the relationship between serum OPG levels and cardiovascular outcomes have employed a similar design and yielded results consistent with ours. This suggests a robust predictive value of serum OPG levels for long-term CVEs in patients with CKD. Lastly, the KNOW-CKD study comprised solely of ethnically Korean patients residing in South Korea. Therefore, extrapolating these findings to other populations should be approached with caution, even though studies from other countries have observed a comparable association between serum OPG levels and the risk of CVEs in the general population.

In conclusion, our findings indicate that elevated circulating OPG levels are associated with adverse cardiovascular outcomes in pre-dialysis CKD patients. Monitoring serum OPG levels could aid in the early detection of CKD patients at elevated risk for future CVEs.

## Methods

### Study population

**Figure 3:**
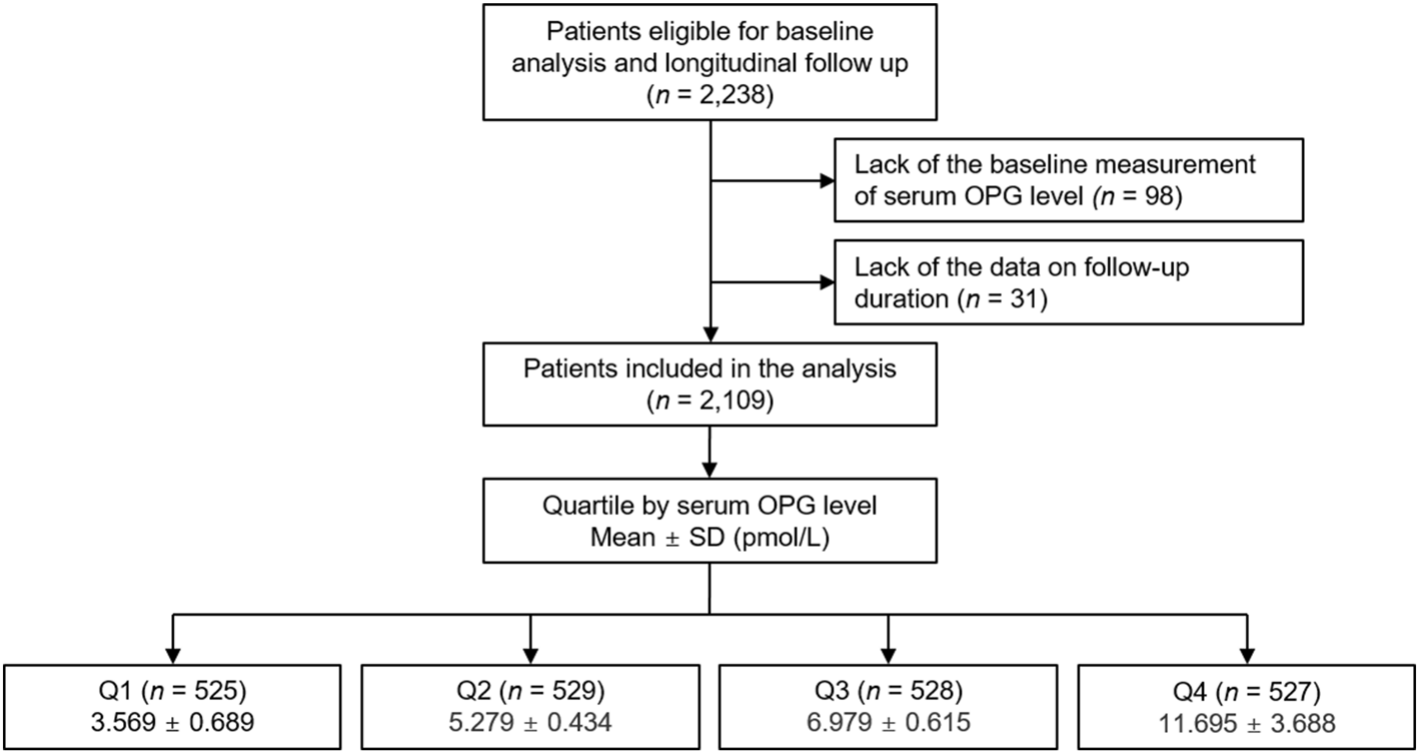
Flow diagram of study participants. *OPG* osteoprotegerin, *Q1* 1st quartile, *Q2* 2nd quartile, *Q3* 3rd quartile, *Q4* 4th quartile, *SD* standard deviation.

The KNOW-CKD (KoreaN Cohort Study for Outcome in Patients With Chronic Kidney Disease)^37^ is a prospective cohort study focused on Korean patients with CKD stages ranging from 1 to pre-dialysis 5. This study was conducted in collaboration with nine tertiary hospitals in South Korea between 2011 and 2016. The study protocol adhered to the Declaration of Helsinki and received approval from the Institutional Review Board of each participating institution: Seoul National University Hospital (1104–089-359, May 25, 2011), Seoul National University Bundang Hospital (B-1106/129–008, August 24, 2011), Yonsei University Severance Hospital (4–2011-0163, June 2, 2011), Kangbuk Samsung Medical Center (2011–01-076, June 16, 2012), Seoul St. Mary’s Hospital (KC11OIMI0441, June 30, 2011), Gil Hospital (GIRBA2553, August 8, 2011), Eulji General Hospital (201105–01, June 10, 2011), Chonnam National University Hospital (CNUH-2011-092, July 5, 2011), and Busan Paik Hospital (11–091, July 26, 2011). Initially, a cohort of 2,238 subjects was established from individuals who provided informed consent. However, after excluding participants without baseline serum OPG measurements (n = 98) and those missing follow-up duration data (n = 31), a total of 2109 subjects remained eligible for further analyses (Fig. 3). Throughout the study, participants were closely monitored, with outcome events recorded by the clinical determination of the investigators at each center. To ensure accuracy, these events were cross-validated by investigators from other collaborating institutions. The median follow-up duration was 7.9 years.

### Data collection from the participants

In accordance with the study protocol, demographic information, anthropometric measurements, and medical histories of the participants were obtained at the time of enrollment. Following an overnight fast, blood and urine samples were collected and subsequently analyzed at the central laboratory (Lab Genomics, Seongnam, Korea). The estimated glomerular filtration rate eGFR was determined using the Chronic Kidney Disease Epidemiology Collaboration equation with creatinine as a reference^38^. For the purpose of measuring the ACR, spot urine samples were collected, preferably as the second void. Echocardiographic evaluations were performed by cardiologists at participating hospitals. These cardiologists were blinded to the clinical data of the participants and carried out the assessments in alignment with the American Society of Echocardiography guidelines^39^.

### Determination of serum OPG levels

The serum OPG concentrations were quantified utilizing an enzyme-linked immunosorbent assay kit (BioVendor R&D, Brno, Czech Republic)^40,41^. Results were reported using the mean values of the duplicate samples. For the samples (n = 3) that had values below the detection range (< 1.5 pmol/L), the value was standardized to 1.5 pmol/L for reporting purposes.

### Exposure and study outcome

Serum OPG concentrations served as the primary exposure metric. Based on these levels, subjects were categorized into quartiles: Q1, Q2, Q3, and Q4 (Fig. 3). The primary study outcome was 3-point MACE, encompassing nonfatal myocardial infarction, nonfatal stroke, and cardiac death. Secondary study outcomes were categorized as 4-point MACE (nonfatal myocardial infarction, unstable angina, nonfatal stroke, and cardiac death) and 6-point MACE (nonfatal myocardial infarction, unstable angina, nonfatal stroke, hospitalization due to heart failure, symptomatic arrhythmia, or cardiac death).

### Statistical analysis

Statistical comparisons of continuous and categorical baseline characteristics based on serum OPG levels were conducted using one-way analysis of variance (ANOVA) and the χ^2^ test, respectively. Kaplan–Meier survival curves were employed to visualize the cumulative incidences of study outcomes, which were subsequently compared using the log-rank test. For participants lost to follow-up, the date of the last visit was considered the censoring date. To determine the independent association between serum OPG levels and the risk of MACEs, Cox proportional hazard regression models were developed. Participants with any missing data were excluded from primary analyses. The models were adjusted as follows: Model 1 reported unadjusted hazard ratios (HRs); Model 2 adjusted for age and gender; Model 3 incorporated adjustments for medical history—specifically, Charlson comorbidity index, primary cause of CKD, smoking status, and medications (e.g., ACEIs/ARBs, diuretics, statins, and antiplatelet/anticoagulant agents)—as well as anthropometric data, namely WHR and SBP. Model 4 further adjusted for baseline laboratory parameters including hemoglobin, albumin, total cholesterol, HDL-C, fasting glucose, 25(OH)D, hs-CRP, eGFR, spot urine ACR, and LVEF. The outcomes of the Cox regression analyses were reported with 95% confidence intervals (CIs) accompanying the HRs. A penalized spline curve illustrated the linear relationship between serum OPG levels (treated as a continuous variable) and the risk of MACEs. To verify the robustness of our findings, we implemented several sensitivity analyses. Initially, participants were grouped by serum OPG levels into tertiles and quintiles instead of quartiles for Cox regression analyses. Next, for the primary study outcome, non-cardiac death events that occurred prior to reaching the study outcome were deemed as a competing risk and thus treated as censoring. Lastly, any missing values in the primary analyses were addressed using multiple imputation and the Cox regression analyses were repeated. Pre-defined subgroup analyses were crafted to explore if the relationship between serum OPG levels and the risk of MACEs differed across specific clinical settings. These subgroups included age (either < 60 or ≥ 60 years), sex (male or female), presence or absence of diabetes mellitus (DM), body mass index (BMI; either < 23 or ≥ 23 kg/m^2^), eGFR (either < 45 or ≥ 45 mL/min/1.73 m^2^), and spot urine ACR (either < 300 or ≥ 300 mg/g). Two-tailed *p* values below 0.05 were deemed statistically significant. All statistical analyses were executed using SPSS for Windows version 22.0 (IBM Corp., Armonk, NY) and R software (version 4.1.1; R Project for Statistical Computing, Vienna, Austria).

### Supplementary Information

Supplementary Information.

## Data Availability

The data collected for this study cannot be shared publicly because they contain information that could compromise the privacy of the research participants. The data are available from the corresponding author upon request.
