# Supplementary material for: Circulating osteoprotegerin levels and cardiovascular outcomes in patients with pre-dialysis chronic kidney disease: results from the KNOW-CKD study
Source: Sci Rep. 2024 Feb 19;14:4136. doi: 10.1038/s41598-024-54335-y (PMC10876961; doi:10.1038/s41598-024-54335-y)
Supplement: Supplementary file 1 — Supplementary Information. [file 41598_2024_54335_MOESM1_ESM.docx]

**- Supplementary Information -**

**Circulating osteoprotegerin levels and cardiovascular outcomes in patients with pre-dialysis chronic kidney disease: Results from the KNOW-CKD study**

Sang Heon Suh, M.D., Ph.D.^1^, Tae Ryom Oh, M.D., Ph.D.^1^, Hong Sang Choi, M.D., Ph.D.^1^, Chang Seong Kim, M.D., Ph.D.^1^, Eun Hui Bae, M.D., Ph.D.^1^, Seong Kwon Ma, M.D., Ph.D.^1^, Kook-Hwan Oh, M.D., Ph.D.^2^, Kyu-Beck Lee, M.D., Ph.D.^3^, Jong Cheol Jeong, M.D., Ph.D.^4^, Ji Yong Jung, M.D., Ph.D.^5^, and Soo Wan Kim*, M.D., Ph.D.^1^, on behalf of the Korean Cohort Study for Outcomes in Patients With Chronic Kidney Disease (KNOW-CKD) Investigators

^1^Department of Internal Medicine, Chonnam National University Medical School and Chonnam National University Hospital, Gwangju, Korea

^2^Department of Internal Medicine, Seoul National University Hospital, Seoul, Korea

^3^Division of Nephrology, Department of Internal Medicine, Kangbuk Samsung Hospital, Sungkyunkwan University School of Medicine, Seoul, South Korea

^4^Division of Nephrology, Department of Internal Medicine, Seoul National University Bundang Hospital, Seongnam, Republic of Korea

^5^Division of Nephrology, Department of Internal Medicine, Gachon University Gil Medical Center, Incheon, Republic of Korea

**Corresponding authors**

*Soo Wan Kim, M.D., Ph.D., Department of Internal Medicine, Chonnam National University Medical School, 42 Jebongro, Gwangju 61469, Korea, Tel: +82-62-220-6271, Fax: +82-62-225-8578, Email: skimw@chonnam.ac.kr

**Table of Contents**

Supplementary Figure S1. Kaplan-Meier survival curve showing cumulative incidence of 4-point MACEs based on serum OPG levels

Supplementary Figure S2. Kaplan-Meier survival curve showing cumulative incidence of 6-point MACEs based on serum OPG levels

Supplementary Figure S3. Panelized spline curve of serum OPG level on 4-point MACE

Supplementary Figure S4. Panelized spline curve of serum OPG level on 6-point MACE

Supplementary Table S1. Summary of echocardiographic findings of study participants by serum OPG levels

Supplementary Table S2. HRs for 4-point and 6-point MACE stratified by serum OPG levels

Supplementary Table S3. HRs for 3-point MACE stratified by serum OPG levels, categorized into tertiles and quintiles

Supplementary Table S4. Previous studies on the association of serum OPG levels and adverse cardiovascular outcomes in patients with CKD


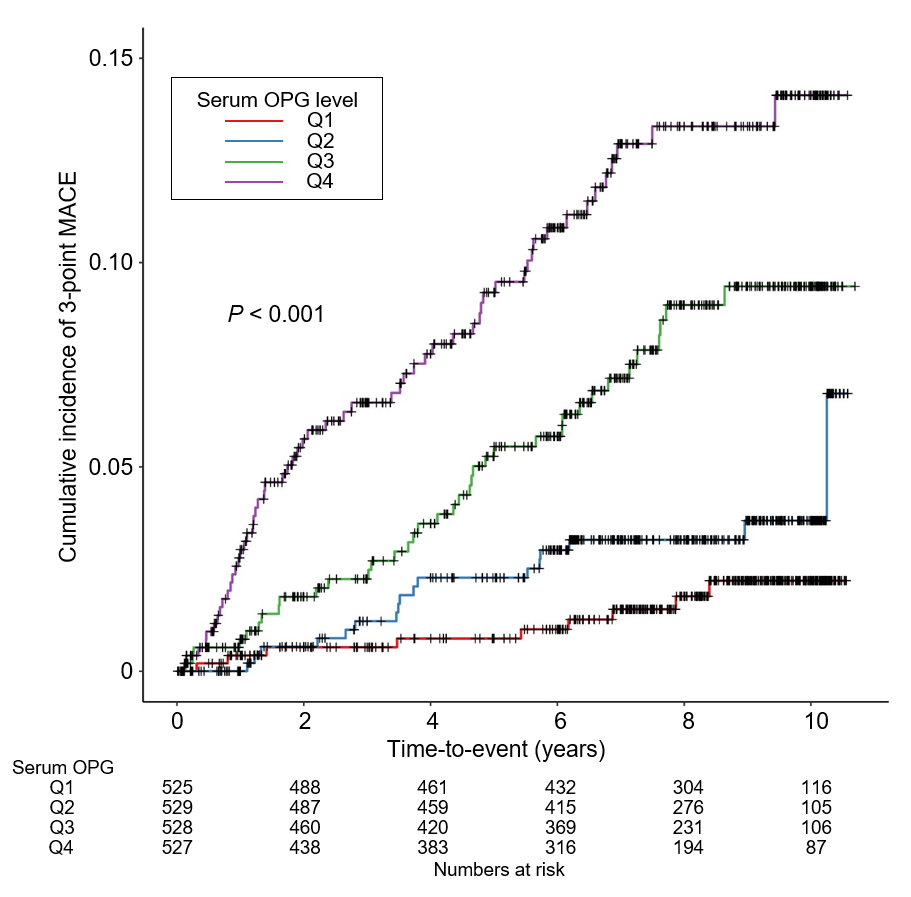


**Supplementary Figure S1. Kaplan-Meier survival curve showing cumulative incidence of 4-point MACEs based on serum OPG levels**

Note: *P* value by log-rank test. Abbreviations: MACE, major adverse cardiac event; OPG, osteoprotegerin; Q1, 1st quartile; Q2, 2nd quartile; Q3, 3rd quartile; Q4, 4th quartile.


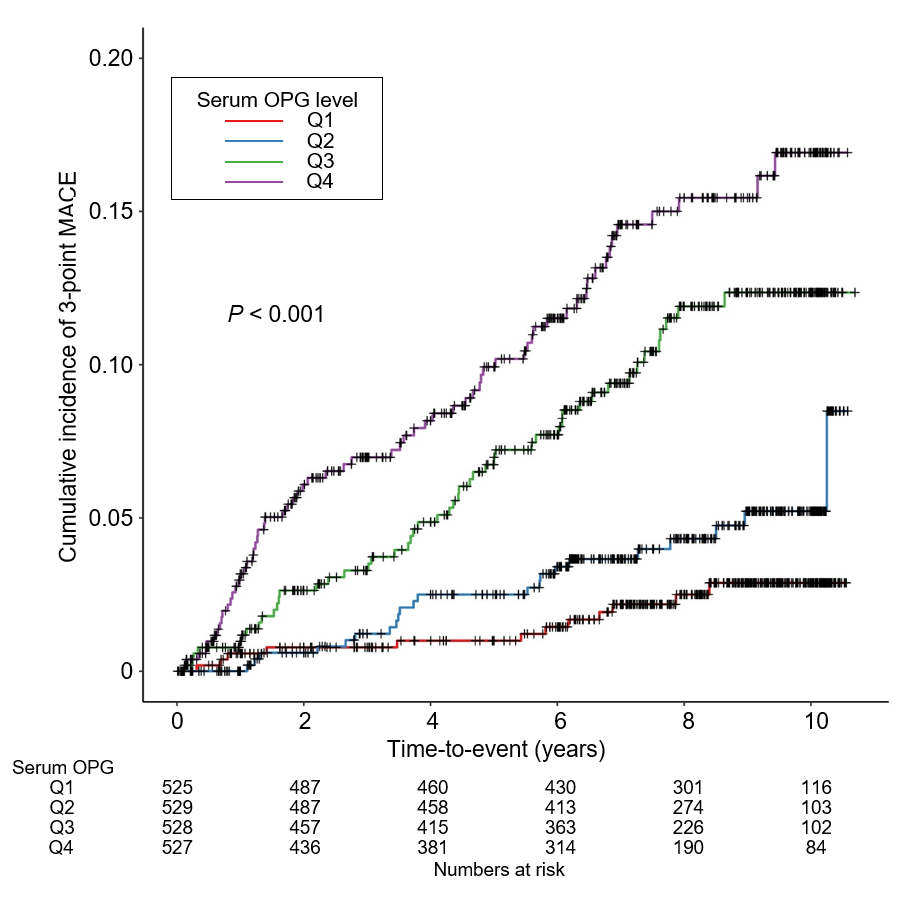


**Supplementary Figure S2. Kaplan-Meier survival curve showing cumulative incidence of 6-point MACEs based on serum OPG levels**

Note: *P* value by log-rank test. Abbreviations: MACE, major adverse cardiac event; OPG, osteoprotegerin; Q1, 1st quartile; Q2, 2nd quartile; Q3, 3rd quartile; Q4, 4th quartile.


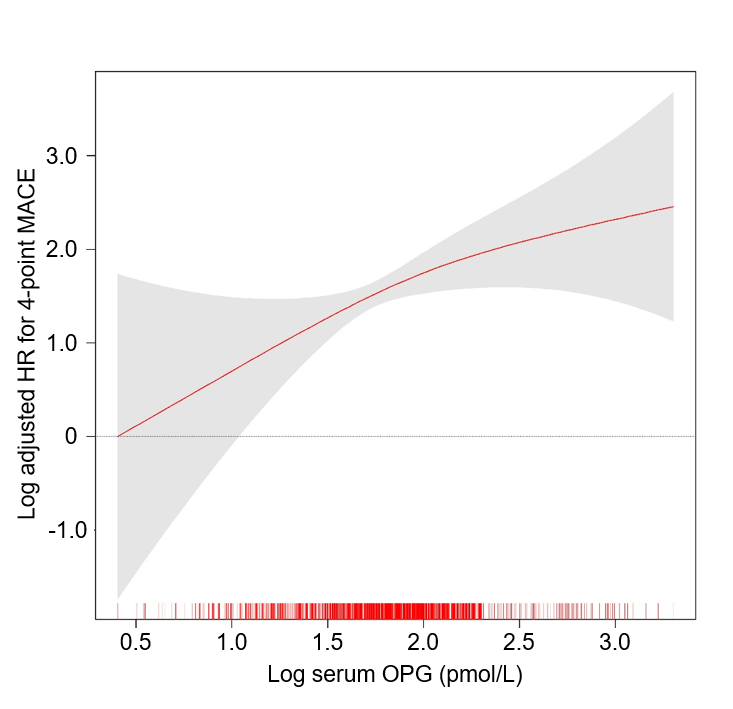


**Supplementary Figure S3. Panelized spline curve of serum OPG level on 4-point MACE**

Note: Adjusted HR of serum OPG level as a continuous variable for 4-point MACE is depicted. The model was adjusted for age, sex, Charlson comorbidity index, primary causes of CKD, smoking status, medication (ACEIs/ARBs, diuretics, statins, antiplatelets/anticoagulants), WHR, SBP, hemoglobin, albumin, total cholesterol, HDL-C, fasting glucose, 25(OH)D, hs-CRP, eGFR, spot urine ACR, and LVEF at the baseline. Abbreviations: HR, hazard ratio; MACE, major adverse cardiac event; OPG, osteoprotegerin.


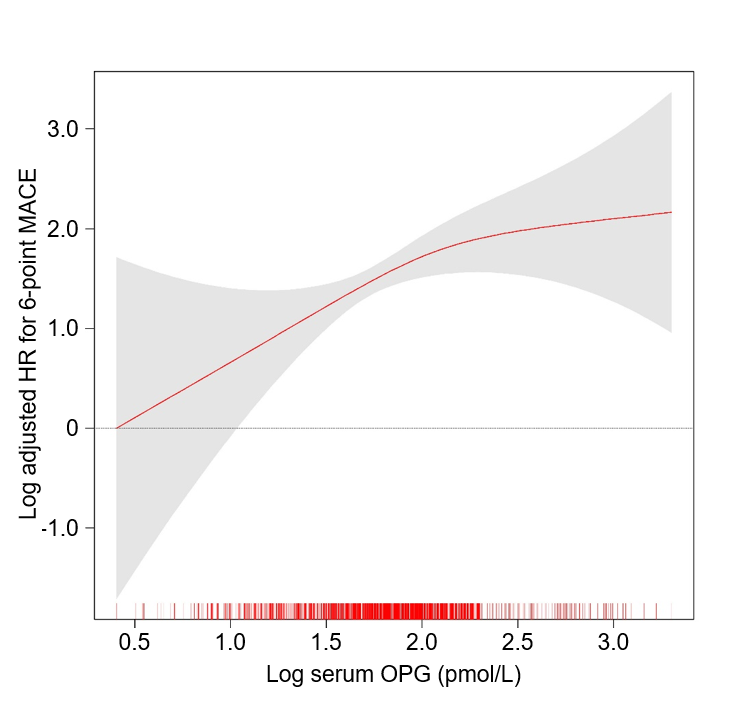


**Supplementary Figure S4. Panelized spline curve of serum OPG level on 6-point MACE**

Note: Adjusted HR of serum OPG level as a continuous variable for 6-point MACE is depicted. The model was adjusted for age, sex, Charlson comorbidity index, primary causes of CKD, smoking status, medication (ACEIs/ARBs, diuretics, statins, antiplatelets/anticoagulants), WHR, SBP, hemoglobin, albumin, total cholesterol, HDL-C, fasting glucose, 25(OH)D, hs-CRP, eGFR, spot urine ACR, and LVEF at the baseline. Abbreviations: HR, hazard ratio; MACE, major adverse cardiac event; OPG, osteoprotegerin.

**Supplementary Table S1. Summary of echocardiographic findings of study participants by serum OPG levels**

|  | Serum OPG levels | | | |  |
| --- | --- | --- | --- | --- | --- |
|  | Q1 | Q2 | Q3 | Q4 | *P* value |
| LVMI (g/m^2^) | 86.047 ± 21.334 | 89.951 ± 21.485 | 95.526 ± 26.385 | 102.790 ± 26.811 | < 0.001 |
| E/e′ | 8.280 ± 2.625 | 9.337 ± 3.161 | 10.361 ± 4.342 | 11.823 ± 4.351 | < 0.001 |
| LVEF (%) | 63.750 ± 5.714 | 64.429 ± 5.855 | 64.169 ± 6.629 | 63.816 ± 6.900 | 0.224 |
| LAD (mm) | 36.351 ± 5.356 | 36.932 ± 5.588 | 38.424 ± 5.908 | 39.347 ± 6.047 | < 0.001 |
| RWMA | 4 (0.8) | 8 (1.5) | 19 (3.6) | 34 (6.6) | < 0.001 |
| Valve calcification | 10 (1.9) | 27 (5.2) | 57 (10.9) | 91 (17.6) | < 0.001 |
| PWT (mm) | 8.849 ± 1.506 | 9.024 ± 1.429 | 9.373 ± 1.605 | 9.814 ± 1.620 | < 0.001 |
| IVWT (mm) | 8.876 ± 1.550 | 9.191 ± 1.540 | 9.514 ± 1.770 | 9.877 ± 1.849 | < 0.001 |
| LVEDD (mm) | 48.852 ± 4.127 | 48.469 ± 4.149 | 48.548 ± 4.851 | 48.744 ± 4.798 | 0.448 |
| LVESD (mm) | 30.808 ± 3.712 | 29.997 ± 3.778 | 30.298 ± 4.570 | 30.487 ± 4.915 | 0.006 |

Note: Values for categorical variables are given as number (percentage); values for continuous variables, as mean ± standard deviation or median [interquartile range]. Abbreviations: E/e′, ratio of the early transmitral blood flow velocity to early diastolic velocity of the mitral annulus; IVWT, interventricular wall thickness; LAD, left atrium diameter; LVEDD, left ventricular end-diastolic diameter; LVEF, left ventricular ejection fraction; LVESD, left ventricular end-systolic diameter; LVMI, left ventricular mass index; PWT, posterior wall thickness; OPG, osteoprotegerin; Q1, 1st quartile; Q2, 2nd quartile; Q3, 3rd quartile; Q4, 4th quartile; RMWA, regional wall motion abnormality.

**Supplementary Table S2. HRs for 4-point and 6-point MACE stratified by serum OPG levels**

|  | Serum OPG levels (pmol/L) | Events, n (%) | Model 1 | | Model 2 | | Model 3 | | Model 4 | |
| --- | --- | --- | --- | --- | --- | --- | --- | --- | --- | --- |
|  |  |  | HR  (95% CI) | *P* value | HR  (95% CI) | *P* value | HR  (95% CI) | *P* value | HR  (95% CI) | *P* value |
| 4-point MACE | Q1 | 9 (1.7) | Reference |  | Reference |  | Reference |  | Reference |  |
|  | Q2 | 17 (3.2) | 2.061  (0.882, 4.815) | 0.095 | 1.406  (0.618, 3.202) | 0.416 | 1.196  (0.515, 2.778) | 0.677 | 1.393  (0.577, 3.364) | 0.461 |
|  | Q3 | 37 (7.0) | 4.302  (1.977, 9.361) | < 0.001 | 2.705  (1.256, 5.827) | 0.011 | 1.983  (0.890, 4.421) | 0.094 | 2.160  (0.908, 5.140) | 0.082 |
|  | Q4 | 57 (10.8) | 7.219  (3.409, 15.288) | < 0.001 | 3.517  (1.612, 7.675) | 0.002 | 2.507  (1.083, 5.802) | 0.032 | 2.571  (1.006, 6.570) | 0.049 |
| 6-point MACE | Q1 | 12 (2.3) | Reference |  | Reference |  | Reference |  | Reference |  |
|  | Q2 | 22 (4.2) | 1.884  (0.903, 3.932) | 0.092 | 1.412  (0.689, 2.891) | 0.346 | 1.271  (0.611, 2.644) | 0.522 | 1.384  (0.644, 2.974) | 0.405 |
|  | Q3 | 49 (9.3) | 4.305  (2.216, 8.363) | < 0.001 | 2.849 (1.462, 5.552) | 0.002 | 2.230  (1.110, 4.480) | 0.024 | 2.471  (1.169, 5.222) | 0.018 |
|  | Q4 | 65 (12.3) | 6.139  (3.208, 11.747) | < 0.001 | 3.233  (1.628, 6.421) | < 0.001 | 2.563  (1.223, 5.371) | 0.013 | 2.686  (1.179, 6.116) | 0.019 |

Note: Model 1, unadjusted model. Model 2, model 1 + adjusted for age and sex. Model 3, model 2 + Charlson comorbidity index, primary cause of CKD, smoking status, medication (ACEi/ARBs, diuretics, statins and antiplatelets/anticoagulants), WHR, and SBP. Model 4, model 3 + adjusted for hemoglobin, albumin, total cholesterol, HDL-C, TG, fasting glucose, 25(OH)D, hs-CRP, eGFR, spot urine ACR, and LVEF at the baseline. Abbreviations: CI, confidence interval; HR, hazard ratio; MACE, major adverse cardiac event; OPG, osteoprotegerin; Q1, 1st quartile; Q2, 2nd quartile; Q3, 3rd quartile; Q4, 4th quartile.

**Supplementary Table S3. HRs for 3-point MACE stratified by serum OPG levels, categorized into tertiles and quintiles**

|  | Serum OPG levels (pmol/L) | | Events, n (%) | Model 1 | | Model 2 | | Model 3 | | Model 4 | |
| --- | --- | --- | --- | --- | --- | --- | --- | --- | --- | --- | --- |
|  |  |  |  | HR  (95% CI) | *P* value | HR  (95% CI) | *P* value | HR  (95% CI) | *P* value | HR  (95% CI) | *P* value |
| 3-point MACE | T1 | 1.5 – 5.0 | 9 (1.3) | Reference |  | Reference |  | Reference |  | Reference |  |
|  | T2 | 5.0 – 7.3 | 32 (4.6) | 4.294  (1.875, 9.831) | < 0.001 | 2.643  (1.228, 5.690) | 0.013 | 2.350  (1.034, 5.345) | 0.042 | 2.657  (1.103, 6.402) | 0.029 |
|  | T3 | 7.3 – 44.2 | 56 (8.0) | 8.296  (3.75, 18.352) | < 0.001 | 3.883  (1.783, 8.456) | < 0.001 | 3.025  (1.274, 7.181) | 0.012 | 3.228  (1.228, 8.484) | 0.018 |
|  | Qu1 | 1.5 – 4.2 | 5 (1.2) | Reference |  | Reference |  | Reference |  | Reference |  |
|  | Qu2 | 4.2 – 5.5 | 8 (1.9) | 1.825  (0.534, 6.233) | 0.337 | 1.330  (0.430, 4.110) | 0.620 | 1.065  (0.333, 3.410) | 0.915 | 1.468  (0.421, 5.126) | 0.547 |
|  | Qu3 | 5.5 – 6.7 | 22 (5.3) | 5.737  (1.969, 16.715) | 0.001 | 3.236  (1.186, 8.833) | 0.022 | 2.793  (1.006, 7.751) | 0.049 | 3.526  (1.135, 10.949) | 0.029 |
|  | Qu4 | 6.7 – 9.0 | 20 (4.7) | 4.565  (1.525, 13.659) | 0.007 | 2.721  (0.970, 7.636) | 0.057 | 1.719  (0.587, 5.034) | 0.323 | 2.237  (0.675, 7.417) | 0.188 |
|  | Qu5 | 9.0 – 44.2 | 42 (10.0) | 10.919  (3.878, 30.741) | < 0.001 | 5.068  (1.830, 14.036) | 0.002 | 3.345  (1.141, 9.807) | 0.028 | 4.079  (1.177, 14.134) | 0.027 |

Note: Model 1, unadjusted model. Model 2, model 1 + adjusted for age and sex. Model 3, model 2 + Charlson comorbidity index, primary cause of CKD, smoking status, medication (ACEi/ARBs, diuretics, statins and antiplatelets/anticoagulants), WHR, and SBP. Model 4, model 3 + adjusted for hemoglobin, albumin, total cholesterol, HDL-C, TG, fasting glucose, 25(OH)D, hs-CRP, eGFR, spot urine ACR, and LVEF at the baseline. Abbreviations: CI, confidence interval; HR, hazard ratio; MACE, major adverse cardiac event; OPG, osteoprotegerin; Qu1, 1st quintile; Qu2, 2nd quintile; Qu3, 3rd quintile; Qu4, 4th quintile; Qu5, 5th quintile; T1, 1st tertile; T2, 2nd tertile; T3, 3rd tertile.

**Supplementary Table S4. Previous studies on the association of serum OPG levels and adverse cardiovascular outcomes in patients with CKD**

| Study | Study Design | Population | Median follow-up | Conclusion |
| --- | --- | --- | --- | --- |
| Scialla *et al.*  (2014) | Prospective cohort in the United States dialysis centers | *n* = 602  (Incident dialysis patients) | 3.4 years | OPG may be risk factors for all-cause and cardiovascular mortality in patients undergoing dialysis |
| Huang *et al.*  (2020) | Meta-analysis | *n* = 2,120  (including 1,723 ESRD patients) | Variable | Elevated OPG concentrations are associated with an increased risk of cardiovascular death in  patients with CKD |
| Marques *et al.*  (2021) | Prospective cohort at Pró-Renal Foundation in Curitiba, Brazil | *n* = 145  (CKD stages 3–5, including ESRD) | 36.5 months | Elevated serum OPG levels were associated with higher all-cause and cardiovascular mortality risk, independent of age, CVD, diabetes, and inflammatory markers, in patients with CKD |

Abbreviations: CKD, chronick kidney disease; CVD, cardiovascular disease; ESRD, end-stage renal disease; OPG, osteoprotegerin.
